# Evolutionary Descent of Prion Genes from the ZIP Family of Metal Ion Transporters

**DOI:** 10.1371/journal.pone.0007208

**Published:** 2009-09-28

**Authors:** Gerold Schmitt-Ulms, Sepehr Ehsani, Joel C. Watts, David Westaway, Holger Wille

**Affiliations:** 1 Centre for Research in Neurodegenerative Diseases, University of Toronto, Toronto, Ontario, Canada; 2 Department of Laboratory Medicine and Pathobiology, University of Toronto, Toronto, Ontario, Canada; 3 Institute for Neurodegenerative Diseases, University of California San Francisco, San Francisco, California, United States of America; 4 Department of Neurology, University of California San Francisco, San Francisco, California, United States of America; 5 Centre for Prions and Protein Folding Diseases, University of Alberta, Edmonton, Alberta, Canada; University of California San Diego, United States of America

## Abstract

In the more than twenty years since its discovery, both the phylogenetic origin and cellular function of the prion protein (PrP) have remained enigmatic. Insights into a possible function of PrP may be obtained through the characterization of its molecular neighborhood in cells. Quantitative interactome data demonstrated the spatial proximity of two metal ion transporters of the ZIP family, ZIP6 and ZIP10, to mammalian prion proteins *in vivo*. A subsequent bioinformatic analysis revealed the unexpected presence of a PrP-like amino acid sequence within the N-terminal, extracellular domain of a distinct sub-branch of the ZIP protein family that includes ZIP5, ZIP6 and ZIP10. Additional structural threading and orthologous sequence alignment analyses argued that the prion gene family is phylogenetically derived from a ZIP-like ancestral molecule. The level of sequence homology and the presence of prion protein genes in most chordate species place the split from the ZIP-like ancestor gene at the base of the chordate lineage. This relationship explains structural and functional features found within mammalian prion proteins as elements of an ancient involvement in the transmembrane transport of divalent cations. The phylogenetic and spatial connection to ZIP proteins is expected to open new avenues of research to elucidate the biology of the prion protein in health and disease.

## Introduction

Prion diseases are fatal neurodegenerative diseases of humans and animals which, in addition to sporadic and familial modes of manifestation, can be acquired via an infectious route of propagation. Prion diseases include sporadic and variant forms of Creutzfeldt-Jakob disease in humans. In sheep, cattle and cervids, prion diseases manifest as scrapie, bovine spongiform encephalopathy, and chronic wasting disease, respectively. The normal, cellular prion protein (PrP^C^) is expressed at high levels in the central nervous system, but can also be found in other cell types within the body. In prion disease, PrP^C^ undergoes a structural transition to its disease-causing form (PrP^Sc^) which possesses profoundly different physicochemical properties [1].

The prion protein is one of the most intensively studied mammalian proteins. Yet surprisingly, the evolutionary origin and physiological function of this protein have remained largely elusive [2], [3]. The function of a protein can sometimes be inferred from genomic investigations which may reveal proteins with similar sequences or sequence modules of known function. Extensive investigations of this kind have provided evidence for PrP-related sequences in most species of the vertebrate lineage [4], [5], [6] and revealed two mammalian paralog sequences encoding for the proteins Doppel (Dpl) and Shadoo (Sho), which together with PrP^C^ constitute the mammalian prion protein family [7]. Despite these advances, no conclusive biological role has emerged for PrP^C^ from the characterization of the additional members of the mammalian prion protein family [8].

Alternatively, by comparing the structural features of particular proteins, otherwise cryptic functional similarities may be inferred. Investigations of PrP^C^ have revealed an unusual dichotomy of its structure, which consists of an extended, largely unstructured N-terminus and a globular and relatively stable C-terminal domain formed by two short β-strands and three α-helices [9]. While proteins with mixed β-strands and α-helices are widespread in nature, aside from Sho and Dpl which share structural features with the N- and C-terminal regions of PrP, respectively, no other protein has been identified which could be used to infer the physiological function of PrP^C^ based on structural similarities. In fact, the ‘prion fold’, present in both PrP^C^ and Dpl, is thus far unique to these two proteins amongst all high-resolution protein structures obtained to date. Extensive evidence has, however, been accumulated which demonstrates that the prion protein can bind a subset of divalent metal cations through two types of histidine-containing motifs embedded within its N-terminal domain [10].

Most proteins do not act in isolation but partner with other proteins to exert their biological roles [11]. Thus, the function of a protein can sometimes be deduced by characterizing its binding partners. Following this ‘guilt-by-association’ logic, we set out to identify the function of the cellular prion protein through a comprehensive interactome investigation. Surprisingly, this work not only revealed a subset of the ZIP family of zinc transporters as novel prion protein interactors, but also shed light on the evolutionary origins of the prion gene family and offers an explanation for the ability of prion proteins to bind divalent cations.

## Results

### Quantitative interactome analyses

Various earlier attempts by us and others had already led to the identification of a few dozen proteins that co-purify with the cellular prion protein under a range of experimental conditions [12]. Often investigations of this kind result in long lists of candidate interactors. The challenge then remains to discriminate specific from unspecific binding partners. Here we incorporated quantitative mass spectrometry based on isobaric tagging of peptides into the workflow to overcome this limitation [13]. Furthermore, investigations were extended to all three members of the mammalian prion protein family to further facilitate discrimination of potential interactors by differential interactome comparison. A murine neuroblastoma cell line (N2a), a cell model widely used for studying prion replication, served as the biological source material [14]. Cells were stably transfected with expression plasmids coding for individual members of the mammalian prion protein family that had been FLAG-tagged in the vicinity of the N-terminus of the mature protein or with a negative control vector [15]. The conceptual choice of both the FLAG-tag and N-terminal attachment site was guided by data documenting that such insertions do not interfere with either PrP^C^ processing or conversion in transgenic animals [16], as well as studies of glycosylation and trafficking in Dpl- and Sho-transfected cells (Coomaraswamy, J., *et al.*, in preparation). To stabilize physiologically relevant interactions, adherent cells were crosslinked by a short treatment with formaldehyde prior to the cell harvest step [17]. Following cell lysis in the presence of detergents, crosslinked protein complexes were affinity-purified based on the presence of the FLAG-affinity tag, trypsinized in solution, and subjected to labeling with isobaric tags for relative and absolute quantitation (iTRAQ) [18]. The samples for the separate prion proteins and the control were mixed and jointly analyzed by tandem mass spectrometry. It should be noted that subsequent to iTRAQ conjugation, the contribution of each sample to the identification of a peptide can be readily calculated by determining the relative intensity of signature iTRAQ mass signals in the relevant collision-induced dissociation (CID) spectra. A comprehensive analysis of samples led to the identification of approximately one hundred proteins, more than thirty of which were observed with iTRAQ signature mass intensity ratios that suggested specific co-enrichment with members of the prion protein family (**Figure S1**, Watts, J.C., *et al*., in revision). Strikingly, two proteins, murine ZIP10 (Slc39a10) and ZIP6 (Slc39a6), which were unequivocally identified with non-overlapping peptides in Dpl, Sho and PrP interactome samples but not in the negative control, represent members of the ZIP family (Zrt-, Irt-like Protein) of transmembrane zinc ion transporter proteins (**Table 1**). In humans and mice, the *slc39a* gene family codes for fourteen distinct proteins (ZIPs 1–14). Amino acid sequence comparisons of the human ZIP proteins argue that ZIP10 and ZIP6, together with their phylogenetically closest paralog ZIP5, constitute a distinct sub-branch within this family [19].

**Table 1:** Quantitative analysis of mouse Dpl, PrP and Sho interactomes identifies metal ion transporters of the ZIP protein family in spatial proximity to all three members of the mammalian prion protein family.

Identified proteins^a^	IPI accession number	Amino acids^c^	Peptides^d^	Unique^e^	% Cov^f^	114^b^ Control	115 Dpl	116 PrP	117 Sho
**Specific binders**									
Dpl	IPI00131622.1	178	8	13	39.1	2.0	92.5	3.0	2.5
PrP	IPI00120793.1	254	5	5	26.8	4.0	20.5	65.1	10.5
Sho	IPI00226455.1	147	3	6	50.4	2.9	11.4	20.5	65.2
ZIP10	IPI00273801.3	833	2	2	4.3	2.4	36.3	16.7	44.6
ZIP6	IPI00469000.4	765	3	3	7.3	3.2	29.9	32.8	34.2
**Unspecific binders**									
Actin	IPI00110850.1	375	21	46	88.5	19.1	21.6	31.8	27.5

a Proteins were sorted into specific versus unspecific binder categories based on their iTRAQ distribution, i.e. proteins were considered unspecific interactors if their derived CID spectra revealed iTRAQ114 to 117 signature mass peak signal intensities which exceeded 10% of combined intensities for all samples including the unspecific control.

b For the calculation of iTRAQ values the intensity of individual peptide associated iTRAQ signature peaks was normalized to combine to 100% per peptide and subsequently averaged. Standard deviations were determined and are listed in **Table S1**.

c Length of precursor molecules prior to posttranslational processing.

d Only CID spectra underlying different peptides were considered i.e. if the same peptide was identified with different charge states or modifications it counted as one hit.

e Total number of unique CID spectra. Please note that the same peptide was only counted more than once if it was identified with different charge states or modifications.

f Percent sequence coverage based on the presence of peptides for which no higher ranked assignment to other proteins could be made.

### Structural and sequence similarities of mammalian prion proteins and ZIPs

**Figure 1:**
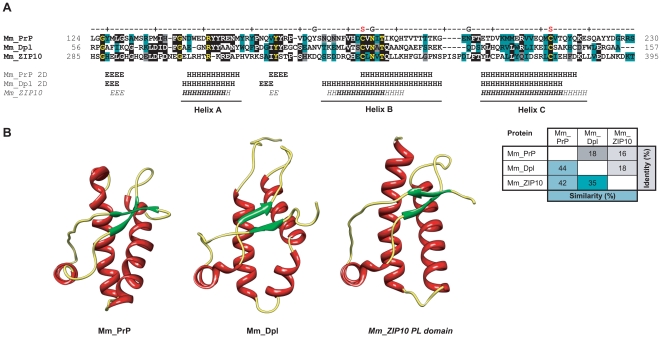
Structural similarity between mouse ZIP10, PrP and Dpl. A, Structural threading of the PL domain within ZIP10 predicts striking resemblance to PrP and Dpl with regard to relative position and order of secondary structure motifs. The secondary structure for the PL domain of ZIP10 was ranked according to the frequency of the prediction in separate threadings (*H* 75–100% and *H* 50–75% for α-helices; the same scale applies to β-sheeted structures “*E* */E*”). Also shown is a matrix of amino acid identities and similarities between depicted sequences of ZIP10, PrP and Dpl. B, Comparison of high-resolution nuclear magnetic resonance structures of PrP (PDB entry: 1ag2) and Dpl (PDB entry:1I17) with a predicted structure for the ZIP10 PL domain. Dark green, grey and black highlights depict conserved, similar and identical residues, respectively. ‘S’ and ‘G’ labels indicate sites of disulfide linkages and glycosylation, respectively.

Intrigued by the presence of the aforementioned ZIP proteins in the dataset (which we had never observed in similarly generated datasets with different bait proteins), these proteins were subjected to an extensive bioinformatic analysis. Surprisingly, the results revealed the presence of a domain within this subset of mouse ZIP proteins that displayed a substantial amino acid sequence similarity to both PrP and Dpl. This initial search was conducted with the SUPERFAMILY database of structural and functional protein annotations (version 1.69) against SCOP (Structural Classification of Proteins) [20] linear hidden Markov models (HMMs; Superfamily model 0037705), a subset of which had been trained on multiple sequence alignments of prion protein ortholog sequences (Superfamily 54098, designated as ‘Prion-like’) [21]. More specifically, a 111-amino acid fragment within the N-terminal extracellular domain of murine ZIP10 (residues 285 to 395) not only showed good general alignment (16% identity, 42% similarity) with the C-terminal globular domain of mouse PrP, but also demonstrated positional agreement of both conserved cysteine residues (which form a disulfide bridge in PrP^C^) and the first N-glycosylation site ‘NxT’ motif found within this domain (**Figure 1A**). Highly similar motifs were also found within the corresponding regions of ZIP6 and ZIP5. Remarkably, the sequence similarity between ZIP10 and PrP in this region is comparable to the similarity observed between Dpl and PrP (18% identity, 44% similarity). No SCOP linear HMM outside the ‘prion-like’ family of HMMs aligned better to this region (from a total of 982 different models in this SCOP release). Indicative of the specificity of this annotation, aside from ZIPs and the aforementioned PrP family members, no other protein among the more than 120,000 mouse and human proteins contained in the LOCATE subcellular localization database (URL: http://locate.imb.uq.edu.au) [22] is recognized to contain a ‘Prion-like’ domain. The E-value for this assignment was 0.011 and therefore had to be qualified as ambiguous. However, SCOP Superfamily algorithms are not optimized to determine remote homologies amongst protein families but instead are largely employed for an initial detection and annotation of protein domains. To further assess the statistical significance of the sequence similarity between PrP and ZIPs in this domain and to ensure that an overemphasis was not placed on outlier sequences, we applied a method dedicated to the detection of remote homologies which uses profile-profile alignments and is embedded in an algorithm that can be initiated from the COMPASS program [23]. A query of this algorithm with the mouse ZIP10 segment 285–385 returned the SCOP40 database entry ‘Prion protein domain’ (designator d.6.1.1) as the only hit (E-value = 4.51e^−4^) which passed the statistical significance threshold of 5e^−3^. The low E-value strongly indicates that sequence similarities are not restricted to a pair of spurious outlier ZIP and PrP sequences and classifies the respective domains within ZIPs and PrP as homologous.

Proteins of common evolutionary origin are frequently characterized by the presence of similar folds. Well-studied examples include proteins of the immunoglobulin and fibronectin type III superfamilies. Such proteins are often the result of divergent evolution during which they have accumulated differences in their primary structure but continue to sustain similar folds. The comparison of protein structures therefore constitutes an orthogonal approach in studying the relationship of proteins. Whereas a multitude of high-resolution structures of prion proteins from many species have been solved, similar information is currently not available for metal ion transporters of the ZIP family.

We employed the FFAS03 fold and function assignment server [24] to carry out a search for any deposited protein structure that would allow (i) a meaningful threading of the ‘prion-like’ (PL) domain in ZIPs 5, 6 or 10 onto any structure template, and (ii) would meet the stringent threshold criteria for significant homology matches embedded in the underlying FFAS03 fold and function assignment algorithm. The published structures of the prion protein and its paralog Doppel were the only structures that fulfilled both of these criteria. More specifically, the profile-based sequence alignment revealed prominent sequence similarities between mouse PrP, Dpl and ZIP10 (**Figure 1A**), which allowed the threading of the sequence of the PL domain of ZIP10 onto the structures of both PrP and Dpl (**Figure 1B**).

The FFAS03 structural alignment score for the ZIP10 PL domain (murine ZIP10 residues 285–395 achieved statistical significance for an alignment with the structure of *Xenopus laevis* PrP (PDB entry: 1xu0) [25] with a value of −9.6 (and a threshold of −9.5 indicating less than 3% false positives). Similarly, the ZIP5 PL domain (murine ZIP5 residues 96–212) also generated a FFAS03 structural alignment score with the same PrP structure in the statistically significant range (with a value of −10.3). Consistently, the models with the strongest scores were based on alignments of PL domains of ZIP proteins with PrP or Dpl structures and all attempts to align the ZIP PL domain to structural templates outside the prion protein family produced insignificant fits. The PL domain of ZIP10 (and also of ZIP5 and ZIP6, not shown) is predicted to contain a structural arrangement very similar to that of PrP or Dpl with three α-helices and possibly a small β-sheet composed of two short β-strands (**Figure 1B**). The two C-terminal α-helices of the ZIP5, ZIP6, and ZIP10 PL domains (helices B and C) are predicted to be stabilized by a disulfide bridge as is the case with the structures of PrP and Dpl. The root mean square deviation (RMSD) between backbone carbon atoms of the nuclear magnetic resonance (NMR) structures for PrP and Dpl is 3.7 Å (as determined by the DaliLite server [26]). Surprisingly, the RMSD between the predicted structure of the ZIP10 PL domain and the PrP and Dpl structures returned even lower values of 2.6 Å and 2.9 Å, respectively, indicating that the primary structure of the ZIP10 PL domain is highly compatible with the basic prion protein fold. Consequently, this observation argues that the secondary structure elements found within the C-terminal domains of PrP and Dpl originated from similar structural features in the ZIP PL domain.

### Biological similarities between PrP and ZIPs

**Figure 2:**
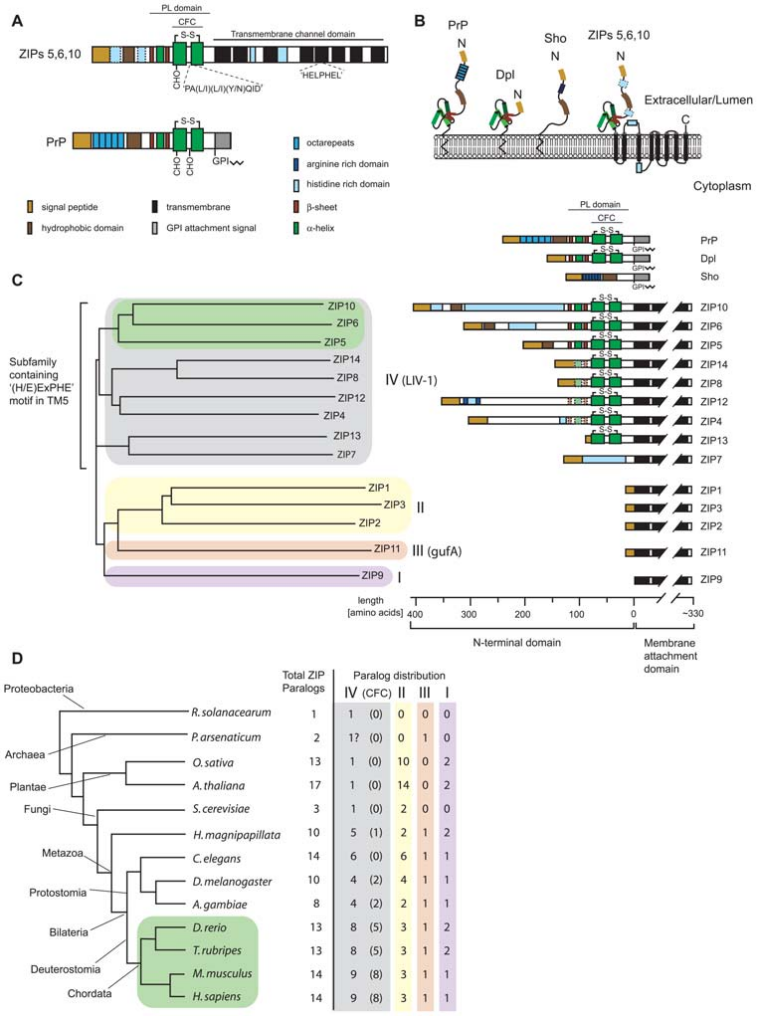
Molecular organization, mode of membrane attachment and phylogenetic relationship of ZIP and prion gene families. A, Schematic drawing depicting the molecular organization of ZIPs 5, 6, 10 (only consensus features shown) and PrP. B, Comparison of orientation and membrane topology of mammalian prion family proteins, and a consensus ZIP5/6/10 molecule. C, Tree diagram depicting the human ZIP family of zinc metal ion transporters. The ZIP protein family in humans (and mice) consists of fourteen paralogs which can be grouped into four subfamilies based on sequence similarities (indicated by different background shading) [19]. Green shading indicates the sub-branch of paralog ZIP sequences most similar to prion family gene sequences. The right side of the panel compares the molecular organization of N-terminal domains within human ZIPs. Please note the divergence in lengths and molecular organization of N-terminal sequences which is contrasted by the presence of a relatively well-conserved cysteine-flanked core (present in 8 out of 14 paralogs) and highly conserved transmembrane domains. D, Simplified phylogenetic tree (modeled after [65]; arbitrary branch lengths) and table depicting the wide distribution of ZIP sequences in most organisms. Green shading indicates the phylogenetic branch of chordates with widespread existence of prion gene ortholog sequences in their genome. The paralog distribution was deduced from an ortholog alignment of 633 ZIP sequences published by the Sanger Institute (TreeFam release 7.0, http://www.treefam.org) or was determined by aligning representative ZIP protein sequences to genomic sequences (proteobacteria, archaea, Cnidaria and fish). The number of ZIP subfamily IV (LIV-1) paralogs containing a cysteine-flanked core (CFC) domain (indicated in brackets) was determined by inspection of sequences for the presence of cysteines that (i) flank a PALLY-like signature motif, and (ii) adhere to CFC consensus distance constraints derived from the multiple alignment of confirmed CFC domains: the cysteine-to-cysteine distance and the cysteine-to-transmembrane attachment site distance. Please note that ZIP proteins harboring CFC sequences N-terminal to their transmembrane domains can be found in organisms with relatively primitive body plans such as hydra (*H. magnipapillata*) or protostomia such as the fruitfly (*D. melanogaster*).

We next compared the known biological features of prion proteins and ZIP proteins of the phylogenetic branch comprising ZIPs 5, 6 and 10 (**Table 2**). Like PrP^C^, ZIP6 and ZIP10 exhibit widespread expression in biological tissues with high transcript levels in the brain [19]. ZIP6 transcripts can also be found in the testis, the predominant site of Dpl expression. The expression of ZIP5 is more restricted and no ZIP5 transcripts can be found in N2a cells by RT-PCR (Ehsani, S., *et al.*, in preparation), consistent with the absence of ZIP5 peptides in our interactome dataset. Reminiscent of the N-terminal repeat motifs in PrP, ZIPs 5, 6 and 10 are equipped with histidine-rich sequences N-terminal to their PL domains. Also reminiscent of PrP, the cysteine-flanked core (CFC) domains (**Figure 2A**) in ortholog sequences of these ZIPs are more highly conserved than the more N-terminal portions of their extracellular sequences. The diversification of the latter is likely aided by an increased chance of recombination events generally associated with tandem repeat regions and previously suggested for prion sequences from different species [6].

**Table 2:** Comparison of mouse PrP gene family paralogs with ZIPs 5/6/10.

Name		Localization			Length^a^			Expression		Function	
Protein	Gene	Compartment	Mode of membrane attachment	N-terminus	Total	Extra- cellular domain	PL domain to membrane distance	Tissue	N2a	Metal binding/transport	Zebrafish knockout phenotype
mZIP5	*slc39a5*	Plasma membrane	Type-3 TM	Extracellular	535	∼210	∼15	Kidney, liver, spleen, colon stomach, pancreas	No	Zn	nd^b^
mZIP6	*slc39a6*	Plasma membrane	Type-3 TM	Extracellular	765	∼334	∼22	Widespread, prominent in brain and testis	Yes	Zn	Impaired mesoderm formation during gastrulation, altered E-cadherin expression [53]
mZIP10	*slc39a10*	Plasma membrane	Type-3 TM	Extracellular	833	∼407	∼30	Widespread, prominent in brain	Yes	Zn	nd
mPrP	*Prnp*	Plasma membrane	GPI-anchor	Extracellular	254	230	17	Widespread, prominent in brain	Yes	Cu, Zn, Fe	Impaired mesoderm formation during gastrulation, altered E-cadherin expression [48]
mSho	*Sprn*	Plasma membrane	GPI-anchor	Extracellular	147	122	n/a	Brain	Yes	nd^b^	nd
mDpl	*Prnd*	Plasma membrane	GPI-anchor	Extracellular	179	157	14	Testis, heart	No	Cu	nd

a In amino acids. Numbers for ZIP proteins are tentative because the exact N-terminal boundary of their transmembrane domain 1 is not known. N-terminal signal peptides included.

b Not determined.

The PL domains within ZIPs 5, 6 and 10 are part of their extracellular N-terminal domains and, with regard to orientation and relative distance to their downstream membrane anchorage sites, resemble PrP^C^ (**Figure 2A, B, C**; **Table 2**). Both PrP^C^ and the above ZIPs belong to a small group of proteins known to be able to concomitantly bind multiple divalent metal ions through histidine-containing motifs embedded in N-terminal repeat sequences [19], [27]. While it is unknown whether PrP^C^'s metal binding ability primarily serves a purpose for sensing, scavenging, or transport of divalent cations *in vivo*, roles of the prion protein in the cellular response to copper-induced oxidative stress [28] and zinc homeostasis [29], [30] have been proposed before, and members of the ZIP protein family are well-characterized in their ability to transport zinc and other divalent metals across membranes [31]. Finally, ZIPs 5, 6 and 10 reside, like PrP^C^, in the plasma membrane and are expected to facilitate import of extracellular zinc into the cytoplasm [19], [31]. Taken together, this context analysis uncovered multiple additional commonalities between ZIP proteins and PrP^C^ consistent with the interpretation that these molecules are evolutionarily related.

### Convergent evolution versus common evolutionary origin

**Figure 3:**
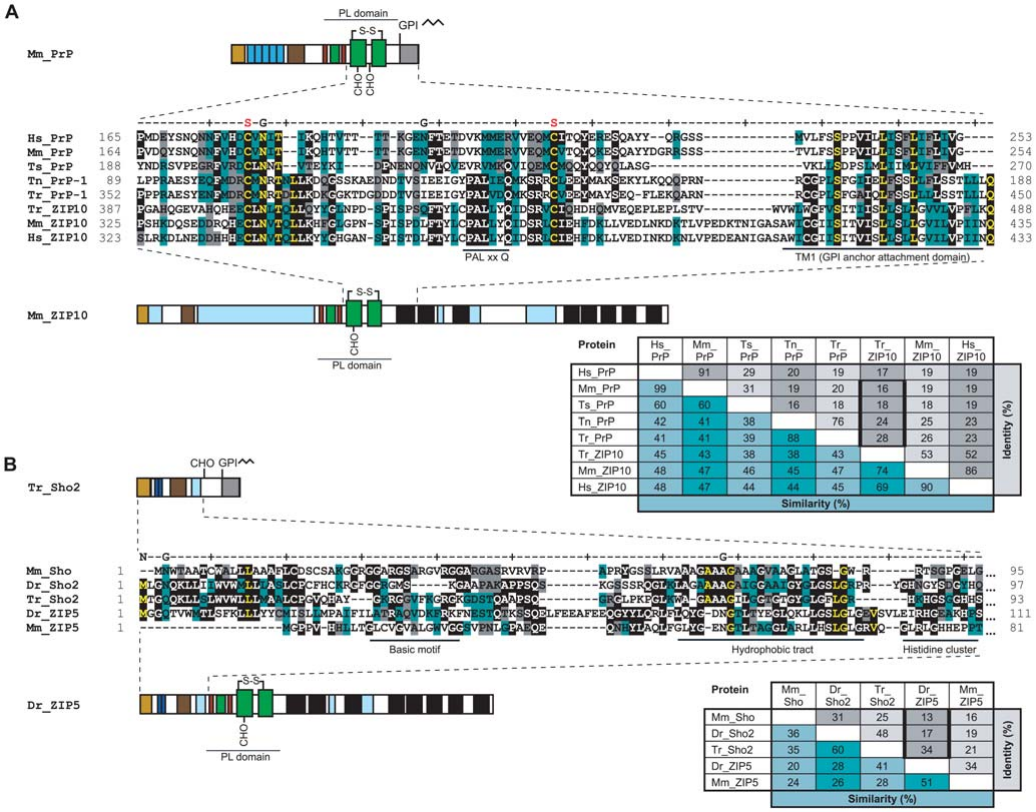
Sequence evidence for common origin and divergent sequence evolution of members of ZIP and prion protein families. A, Multiple sequence alignment of PrP globular domain with ZIP10 PL of selected ortholog sequences. B, Multiple sequence alignment of N-terminal Sho and ZIP5 sequences from pufferfish, zebrafish and mouse. Please note the greater divergence of ZIP10 and PrP in human (Hs: *H. sapiens*) and murine (Mm: *M. musculus*) sequences relative to the respective sequence pairs in turtle (Ts: *T. scripta*) and pufferfish species (Tn: *T. nigroviridis* and Tr: *T. rubripes*). Highlight colors used in the alignments are as in Figure 1. ‘N’, ‘S’, ‘G’ and ‘C’ labels indicate N-termini, disulfide linkages, glycosylation sites and C-termini, respectively. Colors used in the schematic drawing are as in Figure 2A. For full-length multiple alignments of a subset of these and related sequences, please see Figure S2.

It is well-established that similar secondary and tertiary protein structures can evolve independently, as is the case with structural similarity of prokaryotic subtilisin and eukaryotic chymotrypsin [32]. However, instances of convergent evolution at the protein sequence level are rare. Whenever reported, observations appear restricted to convergent adjustments of individual amino acids rather than independent evolutionary inventions of extensive blocks of similar primary structure. A frequently cited case represents the independent adaptation of the protein lysozyme to the acidic milieu found in the digestive tracts of unrelated species [33]. To investigate whether the similarities between PrP and ZIP10 constitute a case of convergent evolution, we analyzed ortholog sequences of both PrP and ZIP10 across a wide range of species within the chordate lineage. This was based on the conception that a divergent trend in sequence similarity would be indicative of evolution from a common phylogenetic ancestor. The opposite scenario, in which the highest sequence similarity is seen in phylogenetically distant species, would constitute evidence for convergent evolution between PrP^C^ and ZIP10 sequences. PrP-related sequences can be found in all mammals and most species of the vertebrate lineage. In contrast, ZIP proteins date back much further and are highly conserved throughout evolution. Related sequences can be found in all kingdoms of life, including bacteria and plants (**Figure 2D**). We therefore compared PrP and ZIP10 ortholog sequences from mammals and fish. Alignment of PrP and ZIP10 sequences from pufferfish (*Takifugu rubripes*) revealed multiple additional amino acid residues conserved between fish ZIP10 and PrP-1, primarily in highly conserved sequence positions (28% sequence identity between pufferfish sequences compared to 16% identity observed in the respective murine sequences) (**Figure 3A**). Consistent with the structural threading (**Figure 1**), small gaps in the alignment of pufferfish sequences corresponded to predicted loops between helices B and C, and the region between helix C and the signal peptide for GPI anchor attachment in PrP (equivalent to the first transmembrane domain in ZIP proteins). Furthermore, a highly conserved ‘PALxxQ’ motif noted by Taylor *et al.* [19] is present within the PL domain of ZIP10 orthologs but is not found in mouse or human PrP sequences. Interestingly, both pufferfish and *Tetraodon nigroviridis* PrP-1 sequences contain this motif (PAL(V/I)(D/E)Q) in perfect positional agreement with the ZIP10 sequence. PrP sequences from turtle (*Trachemys scripta*) (**Figure 3A**), chicken (*Gallus gallus*) and frog (*Xenopus laevis*) (**Figure S3**), organisms that lie between fish and mammals on the evolutionary scale, gave intermediate percent identity values when aligned with ZIP10 sequences (e.g. 18% sequence identity between turtle PrP and pufferfish ZIP10). The elevated sequence identity observed between ZIP10 and PrP protein sequences from fish argues against convergence as an explanation for the similarity of the ZIP and PrP PL domain sequences. Instead, this analysis suggests that PrP descended from a progenitor in the more ancient ZIP family of zinc ion transporters. Another alternative to this parsimonious explanation would be a viewpoint favoring a reverse phylogenetic relationship between these gene families, with PrP as the progenitor. However, here it would be necessary to posit that prion gene sequences disappeared independently in most kingdoms of life except in chordates.

## Discussion

Multiple lines of evidence place a ZIP5/ZIP6/ZIP10-like ancestor gene at the root of the PrP gene family. No single evidence we uncovered is on its own sufficient to firmly establish the phylogenetic relationship between ZIP and prion genes. However, the many orthogonal and corroborating pieces of evidence we collected and, equally important, the absence of any conflicting observations, cumulatively support this conclusion (**Table S2**, see also discussion points below). Beyond explaining the origin of prion genes in the vertebrate lineage, this finding provides a rationale for structural and biological features within modern-day prion proteins as remnants of an ancient involvement in the sensing and/or import of metal ions from the extracellular milieu.

### Protein-protein interactions amongst mammalian prion proteins and ZIPs

The evolutionary relationship between ZIP and prion proteins was discovered in this study on the basis of an interaction between ZIP6 or ZIP10 and PrP, Dpl, or Sho in N2a cells. The fact that these molecules are phylogenetically related and likely possess similar globular folds in their extracellular domains (except Sho which lacks the folded domain) suggests that there may be an inherent ability of proteins containing the prion protein fold to interact with each other. In support of this idea, the ability of PrP^C^ to bind to PrP^Sc^ during prion replication implies that under certain circumstances PrP may be able to recognize itself. It was even reported that Cu^2+^ and Zn^2+^ ions could modulate the self-association of PrP by binding to the octarepeat region and do so in a collaborative fashion despite their different binding affinities [34], [35]. Furthermore, it has been suggested that a direct interaction between PrP^C^ and Dpl may explain the ability of PrP^C^ to protect against Dpl-induced neurotoxicity [36]. Our observation that ZIP10 and ZIP6 co-purified not just with FLAG-PrP and FLAG-Dpl but also with FLAG-Sho, as evidenced by strong iTRAQ117 signals in the CID spectra which led to the identification of ZIP10 and ZIP6 (**Figure S1**), requires a different explanation because FLAG-Sho does not harbor a PL domain. One possibility is that the interaction between Sho and ZIP6 or ZIP10 is indirect (i.e. Sho may bind to a protein which interacts with ZIP6/ZIP10, but does not itself directly interact with the ZIP protein). A second possibility is that multiple binding domains govern the interaction between prion proteins and the ZIP family members. In this scenario, distinct domains present in the N-termini of Sho and PrP^C^ and the C-termini of Dpl and PrP^C^ may be involved in complex formation. Interestingly, the notion of two independent interaction sites between PrP and a hypothetical partner (dubbed “LPrP”) was deduced earlier from transgenic analyses of internally deleted forms of PrP [37].

### N-terminal duplication versus PL domain insertion

While it is generally accepted that the *Prnd* gene, which encodes Dpl, originated from a duplication of the *Prnp* gene, a conclusive phylogenetic link between *Prnp* and *Sprn* (the gene that encodes Sho) has not yet been established. In fish, the genes encoding PrP-1 and Sho-2 are located next to each other, which is indicative of a possible phylogenetic relationship [38]. Furthermore, there is strong sequence similarity between the hydrophobic domains of the two proteins, and Sho has been shown to possess PrP^C^-like protective abilities [8]. In light of the observation that the interactomes of Sho and PrP^C^ in N2a cells are strikingly similar (Watts, J.C., *et al.*, in revision), a phylogenetic relationship between *Sprn* and *Prnp* seems increasingly likely. One evolutionary model has suggested that *Prnp* is derived from *Sprn* by means of an ancient gene duplication event [38]. In this model, *Prnp* acquired the sequences that code for the α-helical C-terminal domain present in modern PrP sequences following its divergence from *Sprn*. Ortholog sequence alignments and structural threading data described in this report suggest that this sequence came from the ancestral protein that gave rise to modern-day ZIP5, 6 and 10.

**Figure 4:**
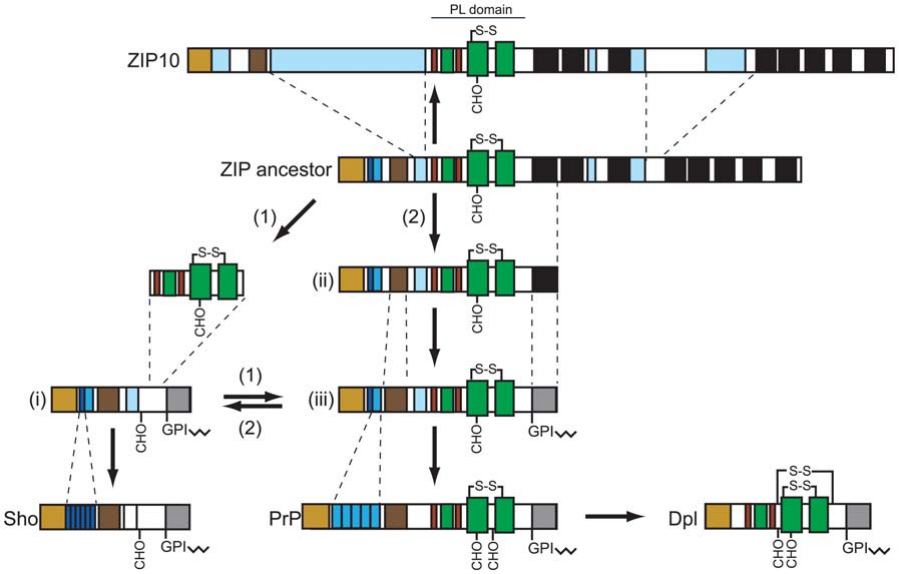
Models depicting evolutionary origin and topology of members of prion protein family, hypothetical ZIP ancestor and ZIP10 transporter. Cartoon depicting the emergence of members of the prion gene family from ZIP ancestor gene(s): (i) hypothetical Sho ancestor gene, (ii) prion gene founder and (iii) evolutionary intermediate prion gene family ancestor. Alternative hypotheses for the origin of prion genes: (1) insertion of ZIP ancestor-derived PL domain into Sho ancestor molecule; or (2) duplication of N-terminal ZIP ancestor fragment followed by expansion of hydrophobic domain and differentiation of former transmembrane domain into signal peptide for attachment of GPI anchor. Based on the above models, Sho genes either (1) evolved independently or (2) were themselves derived from a ZIP ancestor. Please note that our current analyses favor model (2).

Two alternative scenarios seem plausible for the origin of modern-day prion proteins: (i) a gene rearrangement may have caused the fusion of the PL domain of a ZIP ancestor gene with a Sho-like precursor molecule, thereby giving rise to a PrP founder gene (**Figure 4**). This model, as described above, requires that *Sprn* existed prior to *Prnp*. Alternatively, (ii) the complete *Prnp* gene, including promoter elements and sequences N-terminal to the PL domain, could have originated from a ZIP ancestor molecule. If the latter had occurred, similarities between ZIP proteins and PrP should not be restricted to the PL domain but include the respective N-terminal sequences. This model posits that both *Sprn* and *Prnp* are derived from a ZIP-like precursor gene. In support of this theory, the sequences N-terminal to both PrP and ZIP proteins contain similar signal peptides and numerous histidine residues that are involved in binding metal ions. In ZIPs 5, 6 and 10, the histidines are primarily found in clusters whereas in most PrP genes they are found within the context of the tandem octarepeat motifs and a second type of binding site involving histidine residues 95 and 110. A notable exception to this observed segregation represents the amino acid sequence of zebrafish PrP (also known as *D. rerio* PrP-rel-2 or PrP3) which contains extended histidine-rich sequence clusters of the [HX]_n_ variety seen in ZIPs (**Figure S2**) [39], [40]. Although sequence conservation is low N-terminal to the cysteine-flanked core domain between both PrP and ZIP, it should be noted that this region is not well conserved even within orthologs of these protein families, thereby potentially masking evolutionary relationships. Intriguingly, alignment of a Sho-2 sequence from pufferfish showed considerable sequence conservation to ZIP5 from zebrafish (34% identity, 41% similarity) and close examination revealed that (i) a group of basic residues, (ii) the position of a largely hydrophobic segment and (iii) the abovementioned cluster of histidines are shared between these sequences (**Figure 3B**). This sequence alignment falls on the empirical threshold which distinguishes instances of significant similarity from uncertain alignments [41]. Nonetheless, in the context of other similarities detailed above, it further supports the notion that the homology between ZIP and prion protein families extends beyond the globular domain present in PrP and Dpl to include sequences N-terminal to this domain, which are present in Sho (**Figure 4**). To firmly establish which model, if either, is correct, additional *Prnp* and/or *Sprn* ortholog sequences from early chordates will need to be uncovered and compared to ZIP sequences.

### Transmembrane-to-GPI-anchor attachment

Whereas PrP^C^ is inserted into the membrane by the presence of a C-terminal glycosylphosphatidylinositol (GPI) anchor [42], ZIP5, ZIP6 and ZIP10 are type III transmembrane proteins characterized by a relatively large N-terminal extracellular domain, encompassing about half of the molecule's primary structure, and a C-terminal domain harboring up to eight transmembrane spanning segments (**Figure 2A, B**). Precedents exist in other protein families in which some members are inserted into the membrane through transmembrane domains while others employ a GPI anchor for membrane attachment. This dichotomy of membrane attachment modes has, for example, been documented among members of the carcinoembryonic antigen (CEA) protein family [43] and cadherins, a diverse family of GPI-anchored (T-cadherin), single-spanning (classical cadherins and protocadherins) and multi-spanning (7TM-cadherins) transmembrane proteins [44]. In some instances surprisingly small changes in the protein sequence within or in proximity to the first transmembrane domain have been shown to cause a shift to GPI anchor-based modes of membrane attachment [45], [46], [47]. Thus, the GPI anchor signal sequence present in modern PrP may have derived from the first transmembrane domain of a ZIP ancestral molecule (**Figure 4**). In support of this idea, (i) a good alignment is observed between the PrP GPI anchor signal sequence and the ZIP10 TM1 domain for both fish and mammals (**Figure 3A**), and (ii) a consistent distance can be observed between the CFC domains and membrane attachment sites in the two protein families.

### Analyses of genomic sequences

The availability of genomic sequences from the teleost lineage proved highly useful for this work. The presence of multiple copies of prion genes in this vertebrate branch may have helped to preserve original ZIP-like features in a subset of these genes. A phylogenetic linkage of teleost and mammalian prion genes has been established earlier [38] and therefore conclusions drawn from teleost sequence alignments are also relevant for establishing evolutionary relationships of mammalian prions. Interestingly, mouse PrP has recently been shown to partially rescue the phenotypic defects caused by down-regulation of zebrafish PrP-1, arguing that contemporary mammalian and fish PrP molecules have retained some degree of functional similarity throughout their divergent evolution [48].

Whereas the genomic organization of genes encoding for proteins of the prion family has been subjected to close scrutiny previously [49], relatively little is known about regulatory elements and the exon/intron structures of the *slc39a* gene family members. A subset of *slc39a10* ortholog genes and a group of genes from the prion family have in common a first exon containing non-coding regulatory sequence elements and a downstream exon in which a very short segment of non-coding sequence is followed by the respective ATG start codons. The protein coding region of all members of the prion protein family appears to be confined to a single exon. ZIP proteins, in contrast, are encoded by multiple exons. In particular, exon/intron arrangements of ZIP gene sequences encoding for protein sequences upstream of the PL domain are most diverse and may be encoded by a single exon (e.g. mouse ZIP10) or multiple exons with non-conserved exon/intron boundaries across ortholog ZIP sequences. A more complete understanding of genomic rearrangements underlying the emergence of prion sequences from a ZIP ancestor gene should be based on a comparison of all aspects of the respective genomic segments in multiple organisms.

### Structure and function of PL domain

The evolutionary history of the prion protein C-terminal domain has remained a mystery as no other protein for which high-resolution structures have been determined contains the prion protein fold. Our structural threading data suggest that the amino acid sequences within the PL domain region of ZIP5, 6 and 10 are highly compatible with the prion protein fold and the predicted structures are strikingly similar to those previously obtained for PrP and Dpl from various organisms. Since no high resolution structural data currently exists for members of the ZIP family, it will be of great interest to solve the structures of the extracellular domains of ZIP5, 6 and 10 and thereby confirm or refute the structural threading presented here.

The function of the extracellular PL domain within the ZIP family branch containing ZIPs 5, 6 and 10 has yet to be characterized. However, multiple more distantly related paralogs (ZIPs 4, 8, 12, 13 and 14) contain a homologous domain (**Figure 2C**; **Figure S3**) and in the case of ZIP4, a protein that has been genetically linked to a rare recessive zinc deficiency disorder (acrodermatitis enteropathica), a role for N-terminal sequences in the sensing of metal ions has recently been suggested [50]. Interestingly, the abovementioned histidine-rich repeat PrP sequence found in zebrafish has recently been shown to exhibit preferential zinc binding properties [40] (**Figure S2**). Although the preferential binding of copper ions to mammalian PrP has been repeatedly demonstrated, earlier PrP molecules may have harbored different metal ion binding specificities, perhaps reflecting their phylogenetic relationship to zinc transporters. The ability to bind copper has also been described for Dpl, which possesses the prion fold but does not contain an N-terminal histidine-rich domain [51], [52]. Although a knockout of PrP in mice does not result in overt phenotypic deficits, a recent paper has shown that a knockdown of PrP-1 in zebrafish causes a rare gastrulation arrest defect during embryonic development that was linked by the authors to abnormal E-cadherin expression [48]. Interestingly, a strikingly similar defect was observed when ZIP6 (also known as LIV-1) was knocked-down during zebrafish development [53]. Thus, in zebrafish, ZIP6 and PrP-1 may be involved in similar developmental pathways or possess similar functions, possibly related to the regulation of cell adherence and E-cadherin protein levels [48], [53]. For ZIP6, a causal link of this phenotype to zinc transport has been uncovered that involves the zinc-dependent nuclear translocation of the zinc-finger protein Snail, a master regulator of epithelial-mesenchymal transition [53].

### Conclusions

It remains to be determined whether the physical and evolutionary link to ZIP transporters will contribute to efforts towards the elucidation of the physiological functions of members of the prion protein family. It is hoped that a mechanistic understanding of the workings of ZIP transporters as well as high-resolution structures of their extracellular domains may provide insights into the origins of and constraints underlying the conformational changes associated with prion diseases. Additional work could also help to reveal whether membrane-inserted ZIP transporters or N-terminal fragments thereof shed into the extracellular space play a direct role in the manifestation or propagation of prion disease.

## Materials and Methods

### Molecular clones

FLAG affinity tags were inserted before residue 29 of mouse PrP, residue 27 of mouse Dpl, and residue 26 of mouse Sho (all in the pcDNA3 mammalian expression vector) using standard PCR-based mutagenesis techniques. The identity of all constructs was verified by DNA sequencing. The expression of FLAG-tagged bait proteins was confirmed by Western blotting using a FLAG-directed primary antibody (Sigma-Aldrich, Oakville, ON, Canada).

### Cell culture

Mouse neuroblastoma cells (N2a, clone CCL 131, American Type Culture Collection, Rockville, MD) were cultured in Dulbecco's Modified Eagle's Medium (DMEM) containing 10% fetal bovine serum (FBS) and 0.2× penicillin/streptomycin and maintained in a humidified environment in the presence of 5% CO_2_. N2a cells were either transiently or stably transfected with the FLAG-prion constructs or a native pcDNA3 vector (negative control) in OptiMEM using Lipofectamine 2000 (Invitrogen Canada, Burlington, ON, Canada) according to the manufacturer's protocol. For bulk selection of stably transfected cells, cultures were expanded in the presence of 1 mg/mL G418 and maintained at a concentration of 0.2 mg/mL G418.

### Western blotting

Cells were lysed in 0.5% sodium deoxycholate, 1% NP-40, 150 mM NaCl, 50 mM Tris/HCl, pH 8.0 and extracts clarified by centrifugation at 20,000×*g* for 10 min at 4°C. Protein concentrations were determined using the bicinchoninic acid assay (BCA) (Pierce Biotechnology, Rockford, IL, USA). Proteins were separated on 4–12% NuPAGE gels (Invitrogen) and subsequently transferred to polyvinylidene fluoride (PVDF) membranes. Membranes were blocked with 5% milk in Tris-buffered saline containing 0.05% Tween-20 (TBST). Membranes were incubated overnight at 4°C with primary antibody, washed three times with TBST, and incubated with horseradish peroxidase-conjugated secondary antibody (Bio-Rad Laboratories, Hercules, CA, USA) for 2 h at room temperature. Following three washes with TBST, membranes were developed using Western Lightning ECL (PerkinElmer, Woodbridge, ON, Canada).

### *In vivo* crosslinking

Mild formaldehyde crosslinking of N2a cells followed a protocol described before [17]. Briefly, cells grown to confluency were washed with phosphate buffered saline (PBS) and subjected to 15 min crosslinking with 2% w/v formaldehyde in PBS at room temperature. The crosslinking reaction was quenched by incubating cells for 15 min with 125 mM glycine in PBS.

### Affinity purification of bait proteins

Approximately 10^8^ *in vivo* formaldehyde crosslinked cells each of control and FLAG-prion expressing N2a cell lines were lysed in homogenization buffer (50 mM NH_4_Cl, 80 mM Tris, pH 8.0) supplemented with 1× Complete Protease Inhibitor Cocktail (Roche, Palo Alto, CA, USA). To ensure near quantitative extraction of membrane proteins, an equal volume of extraction buffer (20 mM NaCl, 1% deoxycholate, 1% NP-40, 20 mM Tris, pH 8.0) was added, followed by a 30-min incubation and 5-min sonication in a water bath sonicator. Insoluble cellular debris was removed by high-speed centrifugation (100,000×*g*, 1 h). Subsequently, the crosslinked bait protein complexes were immunoaffinity-captured on anti-FLAG-agarose (Sigma-Aldrich, Oakville, ON, Canada). During this step samples were gently agitated on a turning wheel for 12 h, then washed extensively with 0.5 M NaCl, 0.05% SDS, 1% NP-40, 20 mM HEPES, pH 7.3, and detergents removed by a pre-elution wash with 10 mM NH_4_HCO_3_, pH 8.0. Proteins were eluted by acidification with 0.2% trifluoroacetic acid, 20% acetonitrile, pH 2.0.

### Protein reduction, alkylation and trypsinization

Protein-containing fractions were denatured in the presence of 6 M urea, 20 mM NH_4_HCO_3_, pH 8.0, followed by reduction with 1 mM Tris-(2-carboxyethyl)-phosphine for 30 min at 60°C and alkylation with 2.5 mM 4-vinylpyridine for 1 h at room temperature in the dark. Samples were diluted four-fold to ensure that the concentration of urea did not exceed 1.5 M. Tryptic digestion was initiated by the addition of 1% (wt/wt) side chain-modified, TPCK-treated porcine trypsin and allowed to proceed at 37°C for 6 h.

### iTRAQ labeling

Following trypsinization, equal quantities of tryptic peptide mixtures were spiked with 1 pmol of synthetic (Glu1)-Fibrinopeptide B (GluFib) (Sigma-Aldrich) to serve in the downstream analysis as an internal control for the efficiency of individual labeling reactions. Equal labeling with all four reagents was confirmed by the equal intensities of 114∶115∶116∶117 signature peaks upon forced fragmentation of the GluFib [M+2H]^2+^ parent ion at m/z 785.85. Any strong deviation from this ratio would have indicated problems with the labeling reaction or recovery of individual samples prior to the sample mixing step. Individual iTRAQ labeling reagents (Applied Biosystems, Foster City, CA, USA) were reconstituted in ethanol, added to peptide mixtures derived from the tryptic digestion of immunoprecipitation (IP) eluates (Control: iTRAQ 114, Dpl: iTRAQ 115, PrP: iTRAQ 116, Sho: iTRAQ 117) and incubated at room temperature in the dark for 3 h.

### Two-dimensional liquid chromatography

Strong cation exchange (SCX) chromatography was used to achieve peptide fractionation of the complex digest mixture. Samples digested with trypsin were adjusted to 25% acetonitrile and acidified (pH 3.0) by 20-fold dilution in 25% acetonitrile, 20 mM KH_2_PO_4_, pH 3.0. HPLC was carried out using the Ultimate System (Dionex, Sunnyvale, CA, USA) equipped with a microflow calibration cartridge, a Valco injection port and a 180 nL volume UV cell. Separation was achieved on a self-packed 0.5 mm×110 mm Luna SCX (Phenomenex, Torrance, CA, USA) column at a flow rate of 18 µL/min with a steep salt gradient from 0–400 mM NH_4_Cl in 25% acetonitrile, 20 mM KH_2_PO_4_, pH 3.0. Fractions eluted from the SCX column were desalted with C18 Empore (3 M, Minneapolis, MN, USA) stop and go extraction (STAGE) tips [54] and subsequently subjected to nano-flow RP-HPLC using the Ultimate LC system (Dionex) equipped with a nanoflow calibration cartridge at a flow rate of 250 nL/min. Peptides were separated on a 75-µm ID self-packed column containing Proteo C12 reverse-phase matrix (Phenomenex) using a 100-min gradient from 2%–34% acetonitrile in water, with 0.1% (wt/vol) formic acid as the ion-pairing agent.

### ESI-QqTOF mass spectrometry analysis

The analysis of samples by tandem mass spectrometry was essentially done as described before [55]. Briefly, the column effluent was coupled directly via a fused silica capillary transfer line to a QSTAR XL hybrid quadrupole/time-of-flight tandem mass spectrometer (Applied Biosystems; MDS Sciex, Concord, ON, Canada) equipped with a MicroIonSpray source. The progress of each LC/MS run was monitored by recording the total ion current (TIC) as a function of time for ions in the m/z range of 300 to 1800. At 5-s intervals through the gradient, a mass spectrum was acquired for 1 s, followed by one collision-induced dissociation (CID) acquisition of 4 s each on ions selected by preset parameters of the information-dependent acquisition (IDA) method, using nitrogen as the collision gas. Singly-charged ions were excluded from CID selection. The collision energy was adjusted automatically for each CID spectrum using an empirically optimized formula which considers the charge state and m/z value of the precursor ion.

### Database searches

Peak lists for database searching were created using Mascot Distiller (Version 1; MatrixScience, London, UK). Searches were performed using designated MS/MS data interpretation algorithms within Protein Prospector (Version 4.21.3; University of California, San Francisco, CA, USA) [56] and Mascot (Version 2.2; MatrixScience). Modifications considered were oxidation of methionine, phosphorylations of serine and threonine, N-terminal (pyro)Glu and alkylation with 4-vinylpyridine. Searches further considered up to one missed cleavage and charge states ranging from +2 to +4. For a protein to be listed in the data tables it had to be identified by both search algorithms. In the few instances where only two peptides supported the identification of a protein, we required the underlying CID spectra to generate a Mascot score indicating a <5% probability that the match could be considered a random event [57] and further confirmed matches by the peptide sequence tag approach [58] and manual interpretation of spectra. As searches were carried out without species restriction, the correct assignment of matches to mouse entries served as an additional internal control. The mass tolerance range between expected and observed masses used for database searches was ±150 ppm for MS peaks, and ±0.15 Da for MS/MS fragment ions. These relatively large thresholds were used to capture more of the low intense peaks that frequently display broader distribution and thus are assigned with lower mass accuracy. Threshold levels were optimized based on LC/MS/MS datasets of tryptic digests of standard proteins. All samples were searched against the National Center for Biotechnology Information nonredundant database (nrNCBI, release: June 2008) and a ‘decoy’ database in which all entries of the above NCBI database had been inverted. The analysis of iTRAQ data was assisted by the software program ProteinPilot (Applied Biosystems; MDS Sciex). A feature of this software package was used to correct raw iTRAQ ratios for impurity levels of individual reagent lots determined by the manufacturer.

### Multiple sequence alignments

Multiple sequence alignments were obtained using a combination of MAFFT [59] and ClustalX2's implementation of ClustalW2 [60]. Manual adjustments were made to further improve alignments. Sequence identity and conservation were determined using the AlignX feature of Vector NTI Advance 10.3.1 (Invitrogen, Carlsbad, CA, USA) [61].

This algorithm calculates identity values based on the percentage of identical residues among all ungapped positions between sequence pairs. Similarity values are calculated based on the percentage of identical plus similar amino acids among all ungapped positions between pairs. To determine the statistical significance of alignments, the COMPASS program was used [23]. Analyses were based on the ‘SCOP40 iter5’ database, gap opening and extension penalties of 10 and 1, respectively, and the BLOSUM62 substitution matrix.

### Structural threading

The sequences of the PL domains of ZIP5, ZIP6 and ZIP10 were submitted to the FFAS03 fold and function assignment server [24] for structure prediction and structural threading. In order to eliminate threading artifacts based on uncertainties in determining the boundaries of the PL domain, we submitted a number of alternatively truncated sequences to the FFAS03 server. This approach helped to better define the boundaries of the folded region of the PL domain. The FFAS03 server provided a ranked list of structural threading models based on various Protein Data Bank (PDB) entries; invariably the highest scoring models were based on PrP and Dpl structures [25], [62], [63]. We analyzed all sensible models for the position of secondary structure elements and derived a consensus prediction for the secondary structure of the PL domain of ZIP10. The closest model to the consensus prediction was chosen for the corresponding figure. The figures of the PrP and Dpl structures and of the ZIP10 PL domain were produced using the Chimera package from the Computer Graphics Laboratory, University of California, San Francisco (supported by NIH P41 RR-01081) [64].

### Accession numbers

Please refer to **Table S3**.

## Supporting Information

Figure S1 Evidence for specific co-enrichment of ZIP10 and ZIP6 with all three members of the mammalian prion protein family. Comparison of FLAG-affinity chromatography eluates by quantitative tandem mass spectrometry. Side-by-side affinity purified bait protein complexes were trypsinized and subjected to iTRAQ labeling of peptides as follows: iTRAQ114 label: empty vector; iTRAQ115 label: FLAG-Dpl; iTRAQ116 label: FLAG-PrP; and iTRAQ117 label: FLAG-Sho. A, ZIP10 co-purified specifically with the three bait proteins. Collision-induced dissociation (CID) spectrum from ZIP10 derived peptide with amino acid sequence QSTEEIGR ([M+2H]2+, m/z 626.35). Inset: Low mass iTRAQ reporter ion region documenting relative contribution to the identification of this peptide by samples labeled with iTRAQ115 (FLAG-Dpl), iTRAQ116 (FLAG-PrP) and iTRAQ117 (FLAG-Sho) reagents but not negative control sample labeled with iTRAQ114 reagent. B, CID spectrum derived from ZIP6 peptide with amino acid sequence ESASSSEVTSAVYNAVSEGTR ([M+3H]3+, m/z 759.03). iTRAQ reporter ions document that ZIP6 co-purified specifically with the three bait proteins. C, Actin co-purified unspecifically in all four samples including the negative control. CID spectrum derived from actin peptide with amino acid sequence TTGIVMDSGDGVTHTVPIQEGYALPHAILR ([M+4H]4+, m/z 666.35).
(1.49 MB PDF)

Figure S2 Multiple full-length sequence alignment of selected mammalian and teleost ZIP and prion gene sequences. Due to the presence of large insertions found in a subset of depicted sequences, this alignment required manual gapped alignment. A long repeat-motif present only in pufferfish (T. rubripes) PrP but not other sequences included in this alignment is not shown (Tr_PrP amino acids 96-252). Amino acid-specific colors were employed to facilitate the visual comparison of sequences. For descriptive species identifiers, please refer to Figure S3.
(0.13 MB PDF)

Figure S3 Multiple sequence alignment of cysteine-flanked core sequence segment within PL domain. Our data specifically point at a prion ancestor gene in the ZIP family sub-branch containing ZIPs 5, 6 and 10. Please note that all relevant ZIP protein sequences in this branch harbor both flanking cysteines, consistent with the interpretation that these cysteines may engage in a direct disulfide bridge (analogous to the situation in prion proteins). Similarly, the NxT glycosylation motif is shared amongst ZIPs 5, 6, 10 and prion sequences ranging from pufferfish to humans but not found in more distantly related ZIP paralogs. Amino acid-specific colors are as in Figure S2.
(0.11 MB PDF)

Table S1 (0.04 MB PDF)

Table S2 (0.10 MB PDF)

Table S3 (0.11 MB PDF)
