# Supplementary material for: Evolutionary Descent of Prion Genes from the ZIP Family of Metal Ion Transporters
Source: PLoS One. 2009 Sep 28;4(9):e7208. doi: 10.1371/journal.pone.0007208 (PMC2745754; doi:10.1371/journal.pone.0007208)
Supplement: Figure S1 — Evidence for specific co-enrichment of ZIP10 and ZIP6 with all three members of the mammalian prion protein family. Comparison of FLAG-affinity chromatography eluates by quantitative tandem mass spectrometry. Side-by-side affinity purified bait protein complexes were trypsinized and subjected to iTRAQ labeling of peptides as follows: iTRAQ114 label: empty vector; iTRAQ115 label: FLAG-Dpl; iTRAQ116 label: FLAG-PrP; and iTRAQ117 label: FLAG-Sho. A, ZIP10 co-purified specifically with the three bait proteins. Collision-induced dissociation (CID) spectrum from ZIP10 derived peptide with amino acid sequence QSTEEIGR ([M+2H]2+, m/z 626.35). Inset: Low mass iTRAQ reporter ion region documenting relative contribution to the identification of this peptide by samples labeled with iTRAQ115 (FLAG-Dpl), iTRAQ116 (FLAG-PrP) and iTRAQ117 (FLAG-Sho) reagents but not negative control sample labeled with iTRAQ114 reagent. B, CID spectrum derived from ZIP6 peptide with amino acid sequence ESASSSEVTSAVYNAVSEGTR ([M+3H]3+, m/z 759.03). iTRAQ reporter ions document that ZIP6 co-purified specifically with the three bait proteins. C, Actin co-purified unspecifically in all four samples including the negative control. CID spectrum derived from actin peptide with amino acid sequence TTGIVMDSGDGVTHTVPIQEGYALPHAILR ([M+4H]4+, m/z 666.35). (1.49 MB PDF) [file pone.0007208.s001.pdf]

Supplemental Figure 1

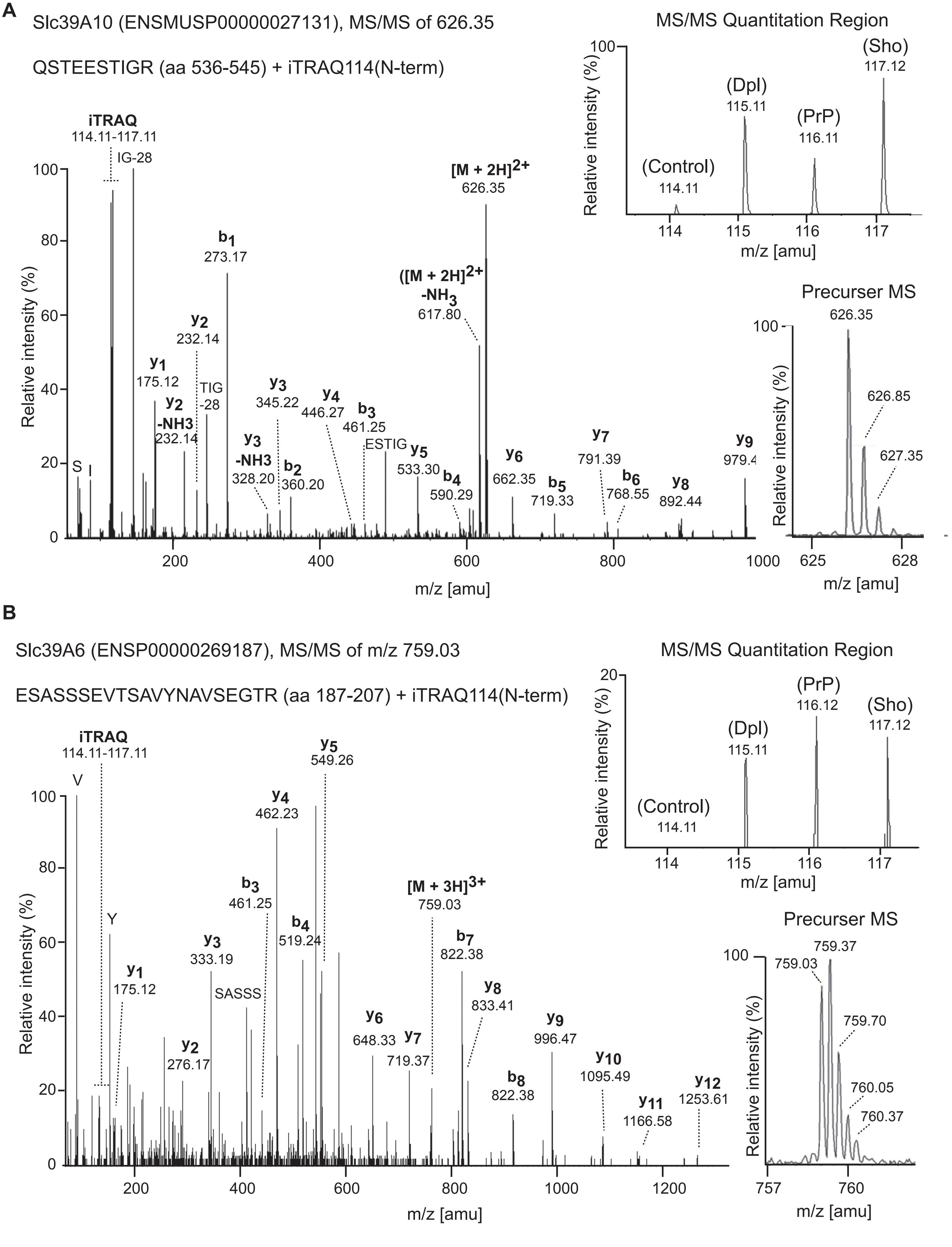

C

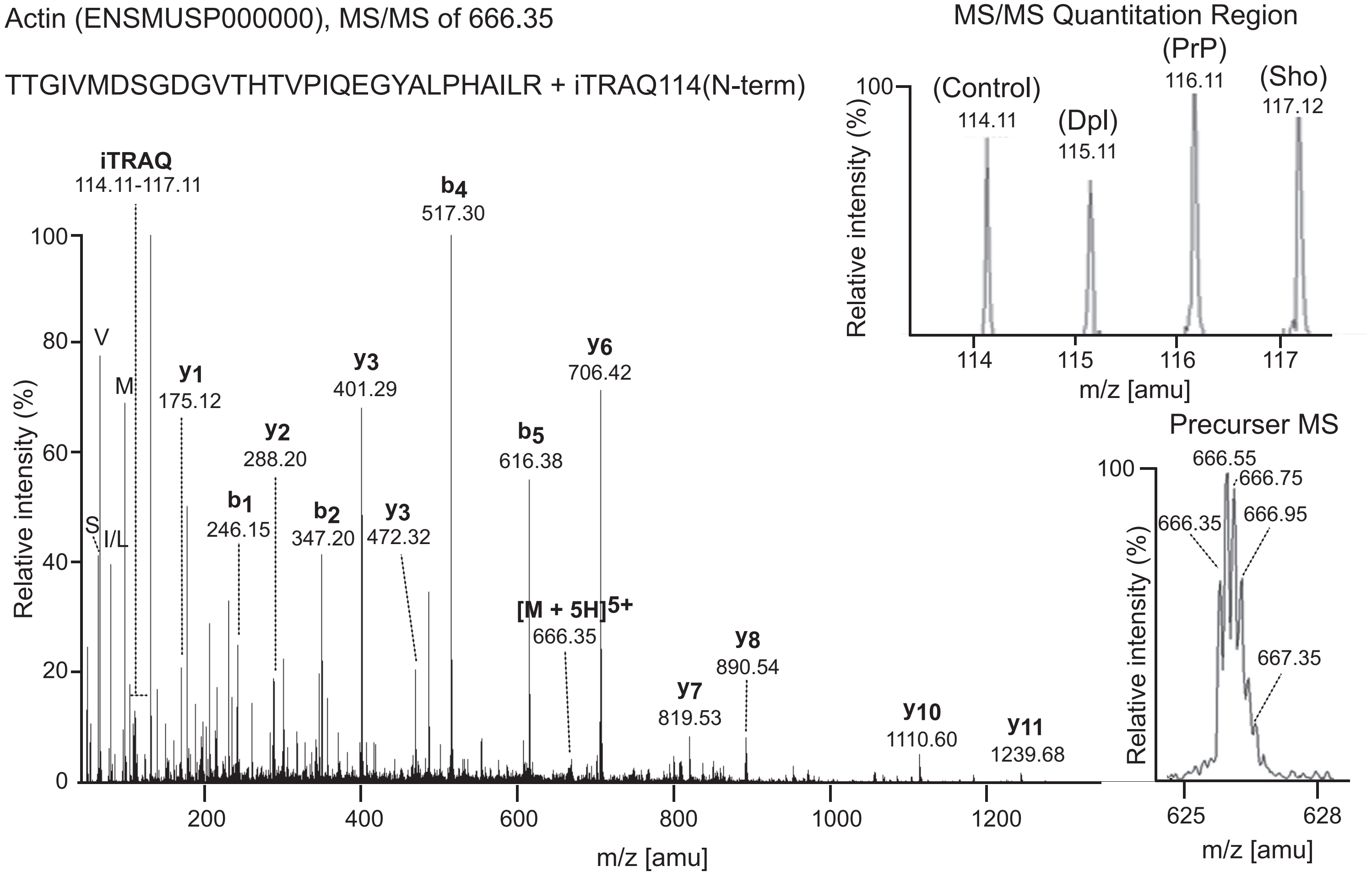
Actin (ENSMUSP000000), MS/MS of 666.35
TTGIVMDSGDGVTHTVPIQEGYALPHAILR + iTRAQ114(N-term)
iTRAQ
114.11-117.11
b4
517.30
V
M
y1
175.12
y3
401.29
y6
706.42
y2
288.20
b5
616.38
b1
246.15
b2
347.20
y3
472.32
S
I/L
[M + 5H]5+
666.35
y8
890.54
y7
819.53
y10
1110.60
y11
1239.68
Relative intensity (%)
m/z [amu]
MS/MS Quantitation Region
(Control)
114.11
(Dpl)
115.11
(PrP)
116.11
(Sho)
117.12
Precurser MS
666.55
666.75
666.35
666.95
667.35
