# Supplementary material for: Evolutionary Descent of Prion Genes from the ZIP Family of Metal Ion Transporters
Source: PLoS One. 2009 Sep 28;4(9):e7208. doi: 10.1371/journal.pone.0007208 (PMC2745754; doi:10.1371/journal.pone.0007208)
Supplement: Figure S2 — Multiple full-length sequence alignment of selected mammalian and teleost ZIP and prion gene sequences. Due to the presence of large insertions found in a subset of depicted sequences, this alignment required manual gapped alignment. A long repeat-motif present only in pufferfish (T. rubripes) PrP but not other sequences included in this alignment is not shown (Tr_PrP amino acids 96-252). Amino acid-specific colors were employed to facilitate the visual comparison of sequences. For descriptive species identifiers, please refer to Figure S3. (0.13 MB PDF) [file pone.0007208.s002.pdf]

Supplemental Figure 2

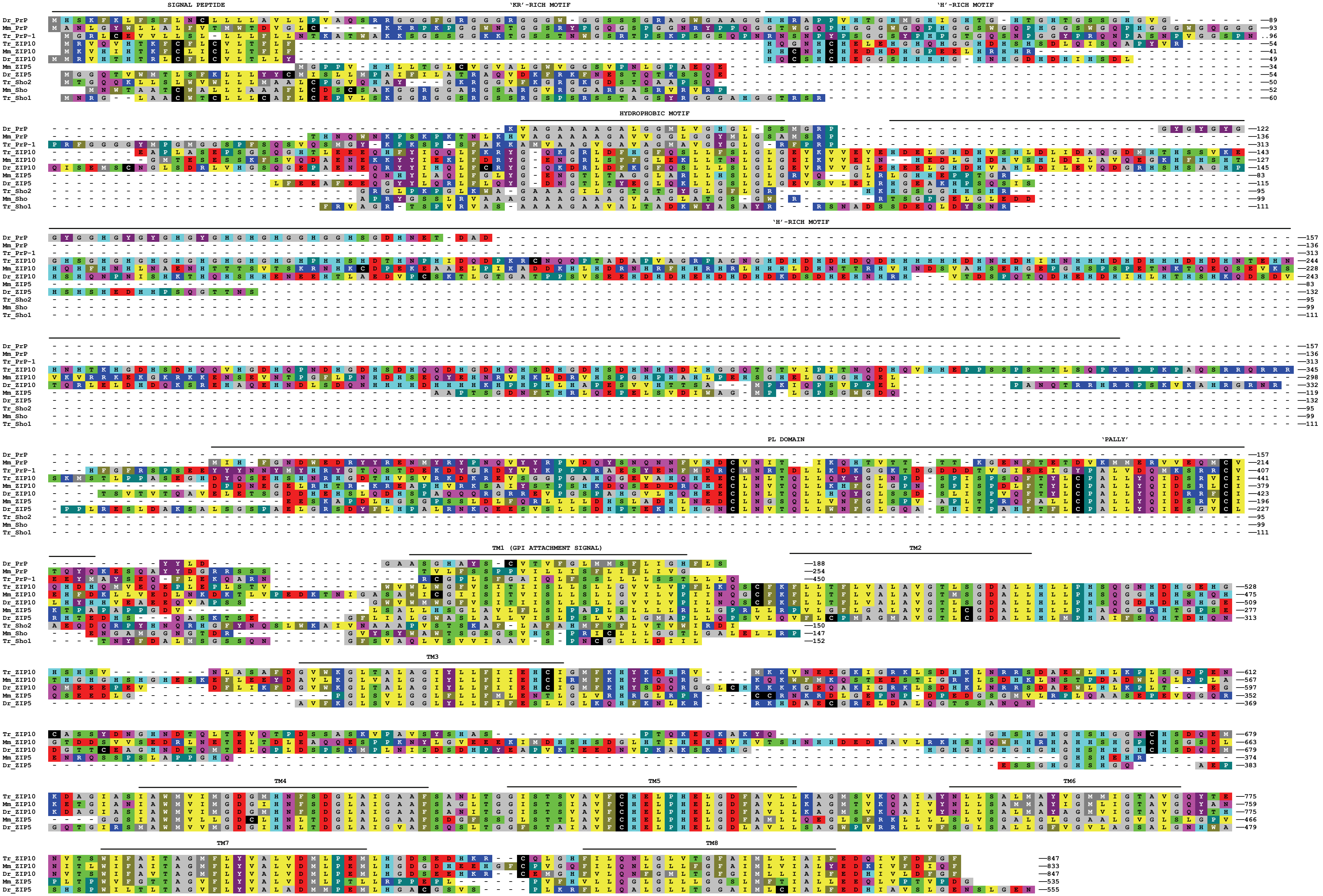

* Please note that an alternative alignment of residues 124 – 136 of Mm_PrP with residues 285 – 297 of Mm_ZIP10 has been presented in Figure 1A since for the structural threading analysis, it was preferred to work with a continuous stretch of primary structure. In this alignment, the emphasis is on evolutionary descent and therefore it was decided that this short sequence segment was best aligned from a phylogenetic perspective.
