# Supplementary material for: Evolutionary Descent of Prion Genes from the ZIP Family of Metal Ion Transporters
Source: PLoS One. 2009 Sep 28;4(9):e7208. doi: 10.1371/journal.pone.0007208 (PMC2745754; doi:10.1371/journal.pone.0007208)
Supplement: Figure S3 — Multiple sequence alignment of cysteine-flanked core sequence segment within PL domain. Our data specifically point at a prion ancestor gene in the ZIP family sub-branch containing ZIPs 5, 6 and 10. Please note that all relevant ZIP protein sequences in this branch harbor both flanking cysteines, consistent with the interpretation that these cysteines may engage in a direct disulfide bridge (analogous to the situation in prion proteins). Similarly, the NxT glycosylation motif is shared amongst ZIPs 5, 6, 10 and prion sequences ranging from pufferfish to humans but not found in more distantly related ZIP paralogs. Amino acid-specific colors are as in Figure S2. (0.11 MB PDF) [file pone.0007208.s003.pdf]

Supplemental Figure 3

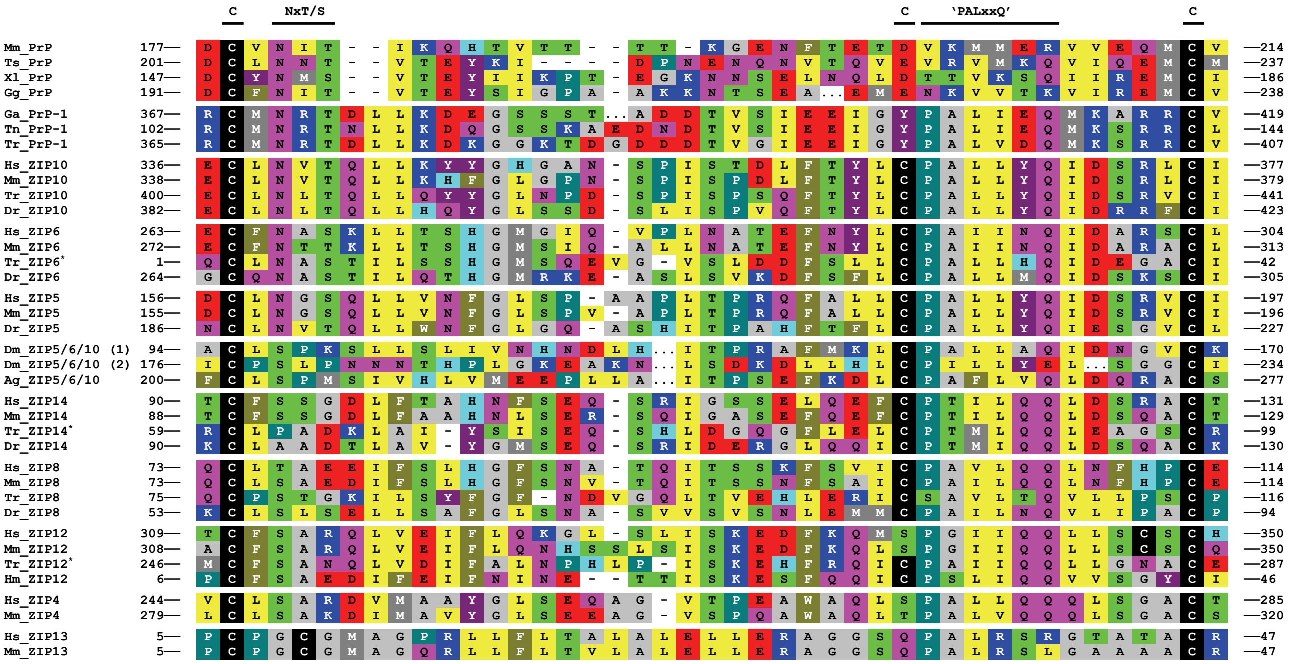

* Complete sequences not available.

Ag → *Anopheles gambiae* (African malaria mosquito)
Dm → *Drosophila melanogaster* (fruit fly)
Dr → *Danio rerio* (zebrafish)
Ga → *Gasterosteus aculeatus* (three-spined stickleback)
Gg → *Gallus gallus* (chicken)
Hm → *Hydra magnipapillata* (hydra)
Hs → *Homo sapiens* (human)
Mm → *Mus musculus* (house mouse)
Tn → *Tetraodon nigroviridis* (spotted green pufferfish)
Tr → *Takifugu rubripes* (Japanese pufferfish)
Ts → *Trachemys scripta* (red-eared slider turtle)
Xl → *Xenopus laevis* (African clawed frog)
