# Supplementary material for: Evolutionary Descent of Prion Genes from the ZIP Family of Metal Ion Transporters
Source: PLoS One. 2009 Sep 28;4(9):e7208. doi: 10.1371/journal.pone.0007208 (PMC2745754; doi:10.1371/journal.pone.0007208)
Supplement: Table S1 — (0.04 MB PDF) [file pone.0007208.s004.pdf]

Supplemental Table 1

Accession number	Symbol	Protein	Conf	Sequence	Modifications	Cleavages	dMass	Prec MW	Prec m/z	Theor MW	Theor m/z	Theor z		Area 114	Area 115	Area 116	Area 117
IPI:IPI00131622.1	DPL	doppel	99	HCDFWLER	iTRAQ4plex@N-term; Pyridylethyl(C)@2		-0.03	1353.61	452.21	1353.64	452.22	3		6.0	89.0	3.4	1.6
			99	VAENRPGAF	iTRAQ4plex@N-term	cleaved F-I@C-term	0.00	1103.58	552.80	1103.58	552.80	2		0.7	94.7	1.7	2.9
			99	VLPSSGGQITEAR	iTRAQ4plex@N-term		-0.01	1457.79	729.90	1457.80	729.91	2		0.1	98.1	1.2	0.6
			99	LDIDFGAEGNR	iTRAQ4plex@N-term		-0.02	1349.65	675.83	1349.67	675.84	2		1.3	94.3	2.6	1.7
			99	VLPSSGGQITEAR	iTRAQ4plex@N-term; Ala->Val@12		0.01	1485.84	496.29	1485.83	496.28	3		1.6	89.9	2.4	6.0
			99	VLPSSGGQITEARVAENRPGAFIKQGR	iTRAQ4plex@N-term; Asn->His@17; Lys->Glu@24	missed R-V@13; missed K-Q@24	0.00	3005.59	752.41	3005.59	752.41	4		3.9	89.9	4.1	2.1
			99	VLPSSGGQITEAR	iTRAQ4plex@N-term; Deamidated(Q)@8; Dimethyl(R)@13		0.06	1486.87	496.63	1486.81	496.61	3		1.0	91.2	4.4	3.3
			99	KVLPSSGGQITEAR	iTRAQ4plex@N-term; iTRAQ4plex(K)@1	missed K-V@1	-0.02	1729.97	577.66	1729.99	577.67	3		1.7	91.0	4.1	3.2
			99	KLDIDFGAEGNR	iTRAQ4plex@N-term; iTRAQ4plex(K)@1	missed K-L@1	-0.04	1621.83	541.62	1621.87	541.63	3		2.4	87.6	6.3	3.7
			99	KVLPSSGGQITEAR	iTRAQ4plex@N-term; iTRAQ4plex(K)@1; HexNAc(T)@11	missed K-V@1	0.02	1933.09	645.37	1933.07	645.36	3		2.5	92.2	3.3	2.0
			99	VAENRPGAFIK	iTRAQ4plex@N-term; iTRAQ4plex(K)@11		0.96	1489.83	497.62	1488.87	497.30	3		0.0	99.4	0.0	0.6
			99	VAENRPGAFIK	iTRAQ4plex@N-term; Lys->Arg@11		-0.05	1372.72	687.37	1372.77	687.39	2		1.0	95.9	1.5	1.6
			92	KVLPSSGGQITEAR	iTRAQ4plex@N-term; Lys->Gln@1	missed K-V@1	0.05	1585.90	529.64	1585.85	529.63	3		3.5	89.0	3.8	3.7
													Average:	2.0	92.5	3.0	2.5
													StDev:	1.7	3.7	1.6	1.5
IPI:IPI00120793.1	PRNP	prion protein	99	HVAGAAAAGAVVGGLGGYMLGSAMSR	iTRAQ4plex@N-term	cleaved R-P@C-term	0.03	2474.29	825.77	2474.26	825.76	3		7.0	25.9	55.7	11.5
			96	QHTVTTTTK	Gln->pyro-Glu@N-term; iTRAQ4plex(K)@9		0.02	1142.62	572.32	1142.61	572.31	2		2.2	24.0	61.0	12.8
			99	PMIHFGNDWEDR	iTRAQ4plex@N-term	cleaved R-P@N-term	0.06	1659.82	554.28	1659.76	554.26	3		6.1	26.8	55.3	11.8
			97	ESQAYYDGR	iTRAQ4plex@N-term		0.00	1231.56	616.79	1231.56	616.79	2		3.4	11.5	76.4	8.6
			99	VVEQMCVTQYQK	iTRAQ4plex@N-term; Pyridylethyl(C)@6; iTRAQ4plex(K)@12		-0.09	1847.86	616.96	1847.95	616.99	3		1.1	14.2	76.9	7.7
													Average:	4.0	20.5	65.1	10.5
													StDev:	2.5	7.1	10.8	2.2
IPI:IPI00226455.1	SPRN	shadoo	99	ICLLLGGTLGALELLRP	iTRAQ4plex@N-term; Pyridylethyl(C)@2		-0.01	2000.19	667.74	2000.20	667.74	3		0.0	6.9	32.4	60.7
			99	VAAAGAAAGAAAGVAAGLATGSGWR	iTRAQ4plex@N-term		-0.04	2198.13	733.72	2198.17	733.73	3		0.0	21.6	18.3	60.1
			98	YGSSLR	iTRAQ4plex@N-term		0.00	825.45	413.73	825.45	413.73	2		0.8	2.3	23.2	73.8
			99	VAAAGAAAGAAAGVAAGLATGSGWR	iTRAQ4plex@N-term; Oxidation(W)@24		-0.01	2214.15	739.06	2214.16	739.06	3		8.0	13.4	15.0	63.5
			99	VAAAGAAAGAAAGVAAGLATGSGWR	iTRAQ4plex@N-term; Val->Asn@14		0.05	2213.19	738.74	2213.14	738.72	3		7.1	12.9	15.4	64.7
			99	VAAAGAAAGAAAGVAAGLATGSGWR	iTRAQ4plex@N-term; Val->Met@14		0.07	2230.21	744.41	2230.14	744.39	3		1.4	11.1	18.8	68.7
													Average:	2.9	11.4	20.5	65.2
													StDev:	3.7	6.5	6.5	5.2
IPI:IPI00469000.4	SLC39A6	ZIP6	99	ESASSSEVTSAVYNAVSEGTR	iTRAQ4plex@N-term		0.01	2274.08	759.03	2274.07	759.03	3		3.7	29.2	35.9	31.2
			95	AGMTVK	iTRAQ4plex@N-term; Dehydrated(T)@4; iTRAQ4plex(K)@6		-0.03	875.48	438.75	875.51	438.76	2		2.2	31.1	31.9	34.8
			99	YDSQLSSNEEK	iTRAQ4plex@N-term; Lys->Gln@11		0.06	1442.69	722.35	1442.63	722.32	2		3.7	29.2	30.5	36.6
													Average:	3.2	29.9	32.8	34.2
													StDev:	0.8	1.1	2.8	2.8
IPI:IPI00273801.3	SLC39A10	ZIP10	99	QSTEESTIGR	iTRAQ4plex@N-term		0.03	1250.65	626.33	1250.62	626.32	2		2.3	39.3	15.7	42.7
			98	NYLGVEEEK	iTRAQ4plex@N-term; Lys->Gln@9		0.07	1223.65	612.83	1223.58	612.80	2		2.5	33.2	17.7	46.5
													Average:	2.4	36.3	16.7	44.6
													StDev:	0.2	4.3	1.4	2.7
IPI:IPI00110850.1	ACTB	actin	99	AGFAGDDAPR	iTRAQ4plex@N-term		0.02	1119.56	560.79	1119.54	560.78	2		27.5	13.1	37.0	22.4
			99	AVFPSIVGR	iTRAQ4plex@N-term	cleaved R-P@C-term	-0.02	1088.63	545.32	1088.65	545.33	2		27.2	13.0	27.9	31.9
			99	AVFPSIVGRPR	iTRAQ4plex@N-term		-0.01	1341.79	448.27	1341.80	448.27	3		24.9	19.8	33.3	22.0
			99	AVFPSIVGRPR	iTRAQ4plex@N-term; Deamidated(R)@9		-0.03	1342.76	448.59	1342.78	448.60	3		25.5	14.2	33.8	26.5
			84	AVFPSIVGRPR	iTRAQ4plex@N-term; Dimethyl(R)@11		0.01	1369.85	457.62	1369.83	457.62	3		28.8	16.8	32.9	21.5
			99	DLTDYLMK	iTRAQ4plex@N-term; iTRAQ4plex(K)@8		0.00	1285.68	643.85	1285.68	643.85	2		18.2	23.6	37.5	20.7
			98	DLTDYLMK	iTRAQ4plex@N-term; Lys->Gln@8		0.02	1141.57	571.79	1141.54	571.78	2		10.6	8.5	30.5	50.4
			99	DLYANTVLSGGTTMYPGIADR	iTRAQ4plex@N-term		0.13	2358.29	787.10	2358.16	787.06	3		11.0	29.3	30.3	29.4
			99	DLYANTVLSGGTTMYPGIADR	iTRAQ4plex@N-term; Dimethyl(R)@21		0.03	2386.23	796.42	2386.20	796.41	3		30.1	18.6	0.0	51.3
			99	DLYANTVLSGGTTMYPGIADR	iTRAQ4plex@N-term; Leu->Pro@2		-0.02	2342.12	781.71	2342.13	781.72	3		5.7	28.2	29.4	36.7
			99	DSYVGDEAQSK	iTRAQ4plex@N-term; Dehydrated(S)@2; Lys->Gln@11		0.04	1323.61	662.81	1323.57	662.79	2		29.3	8.9	29.6	32.2
			99	DSYVGDEAQSK	iTRAQ4plex@N-term; iTRAQ4plex(K)@11		0.04	1485.76	496.26	1485.72	496.25	3		22.5	29.7	31.8	16.0
			99	DSYVGDEAQSK	iTRAQ4plex@N-term; Lys->Arg@11		0.04	1369.67	685.84	1369.62	685.82	2		21.7	10.8	31.2	36.4
			99	DSYVGDEAQSK	iTRAQ4plex@N-term; Lys->Gln@11		0.04	1341.62	671.82	1341.58	671.80	2		21.6	9.4	33.0	35.9
			87	DSYVGDEAQSK	iTRAQ4plex@N-term; Ser->Asp@10; iTRAQ4plex(K)@11		0.01	1513.72	505.58	1513.71	505.58	3		26.7	17.7	34.1	21.6
			92	DSYVGDEAQSK	iTRAQ4plex@N-term; Ser->Val@10; iTRAQ4plex(K)@11		0.01	1497.76	500.26	1497.76	500.26	3		27.3	25.1	28.8	18.8
			99	DSYVGDEAQSKR	iTRAQ4plex@N-term; iTRAQ4plex(K)@11	missed K-R@11	0.04	1641.86	548.29	1641.82	548.28	3		17.9	32.4	44.2	5.5
			96	DSYVGDEAQSKR	iTRAQ4plex@N-term; Lys->Gln@11	missed K-R@11	0.08	1497.76	500.26	1497.68	500.23	3		19.9	30.6	33.0	16.4
			80	EITALAPSTMK	Glu->pyro-Glu@N-term; iTRAQ4plex(K)@11		0.06	1286.76	644.39	1286.70	644.36	2		25.0	12.1	33.5	29.5
			99	EITALAPSTMK	iTRAQ4plex@N-term; Lys->Arg@11		0.01	1332.73	667.37	1332.72	667.37	2		19.8	11.4	33.8	35.0
			89	EITALAPSTMK	iTRAQ4plex@N-term; Lys->Gln@11		0.07	1304.75	653.38	1304.68	653.35	2		20.7	19.2	32.7	27.4
			99	EITALAPSTMKI	iTRAQ4plex@N-term; Lys->Ala@11; Ile->Ala@12	cleaved I-K@C-term; missed K-I@11	0.01	1318.70	660.36	1318.69	660.35	2		18.0	8.7	28.2	45.1
			99	GYSFTTTAER	iTRAQ4plex@N-term		0.00	1275.63	638.82	1275.62	638.82	2		13.6	14.7	36.8	34.9
			99	GYSFTTTAER	iTRAQ4plex@N-term; Dimethyl(R)@10		0.05	1303.71	652.86	1303.65	652.83	2		22.5	6.8	32.6	38.0
			99	HQGVMVGMGQK	iTRAQ4plex@N-term; iTRAQ4plex(K)@11		0.00	1458.77	487.26	1458.77	487.26	3		14.5	30.6	37.5	17.4
			90	HQGVMVGMGQK	iTRAQ4plex@N-term; Lys->Arg@11		0.03	1342.70	448.57	1342.67	448.56	3		11.4	13.7	34.2	40.7
			99	HQGVMVGMGQK	iTRAQ4plex@N-term; Lys->Gln@11		0.05	1314.68	439.23	1314.63	439.22	3		14.2	22.0	31.8	32.0
			99	KDLYANTVLSGGTTMYPGIADR	iTRAQ4plex@N-term; iTRAQ4plex(K)@1		0.04	2630.40	658.61	2630.36	658.60	4		12.5	33.2	28.5	25.8
			99	LDLAGRDLTDYLMK	iTRAQ4plex@N-term; iTRAQ4plex(K)@14	missed R-D@6	0.01	1911.05	638.02	1911.04	638.02	3		40.6	15.9	32.7	10.8
			98	MTQIMFETFNTPAMYVAIQAVLSLYASGR	iTRAQ4plex@N-term		0.07	3396.77	850.20	3396.70	850.18	4		8.9	52.0	28.3	10.8
			99	QEYDESGPSIVHR	iTRAQ4plex@N-term		0.04	1659.84	554.29	1659.80	554.27	3		6.6	42.9	29.0	21.5
			99	SYELPDGQVITIGNER	iTRAQ4plex@N-term		-0.02	1933.97	645.66	1933.99	645.67	3		11.2	41.0	30.4	17.5
			99	SYELPDGQVITIGNER	iTRAQ4plex@N-term; Asn->Arg@14		0.01	1976.05	659.69	1976.04	659.69	3		18.3	20.0	36.3	25.4
			99	SYELPDGQVITIGNER	iTRAQ4plex@N-term; Asp->Pro@6		-0.08	1915.93	639.65	1916.01	639.68	3		5.5	16.6	32.5	45.4
			99	SYELPDGQVITIGNER	iTRAQ4plex@N-term; Deamidated(N)@14; Glu->Arg@15		0.02	1962.05	655.02	1962.03	655.02	3		20.0	23.6	28.9	27.5
			99	SYELPDGQVITIGNER	iTRAQ4plex@N-term; Dehydrated(D)@6		-0.05	1915.93	639.65	1915.98	639.67	3		10.9	13.7	33.6	41.9
			81	SYELPDGQVITIGNER	iTRAQ4plex@N-term; Dehydrated(D)@6; Dimethyl(R)@16		0.01	1944.02	649.01	1944.01	649.01	3		13.0	21.2	41.0	24.8
			99	SYELPDGQVITIGNER	iTRAQ4plex@N-term; Dimethyl(R)@16		-0.01	1962.01	655.01	1962.02	655.01	3		18.6	14.0	37.5	29.9
			99	TTGIVMDSGDGVTHTVPIYEGYALPH	iTRAQ4plex@N-term	cleaved H-A@C-term	-0.04	2873.36	958.79	2873.40	958.81	3		7.7	19.7	30.5	42.0
			99	TTGIVMDSGDGVTHTVPIYEGYALPHAILR	iTRAQ4plex@N-term		0.04	3326.75	832.69	3326.71	832.68	4		24.8	20.5	29.3	25.4
			99	TTGIVMDSGDGVTHTVPIYEGYALPHAILR	iTRAQ4plex@N-term; Dimethyl(R)@30		-0.03	3354.72	839.69	3354.74	839.69	4		31.5	14.5	32.5	21.5
			99	TVLSGGTTMYPGIADR	iTRAQ4plex@N-term	cleaved N-T@N-term	0.03	1781.94	594.99	1781.91	594.98	3		21.1	16.9	34.7	27.2
			99	VAPEEHPVLLTEAPLNPK	Carbamyl@N-term; iTRAQ4plex(K)@18		-0.04	2140.13	714.38	2140.17	714.40	3		1.1	67.1	19.4	12.4
			93	VAPEEHPVLLTEAPLNPK	Formyl@N-term; iTRAQ4plex(K)@18		-0.02	2125.14	709.39	2125.15	709.39	3		43.3	19.8	23.3	13.7
			99	VAPEEHPVLLTEAPLNPK	iTRAQ4plex@N-term; Lys->Arg@18		-0.04	2125.12	709.38	2125.17	709.40	3		20.6	24.9	33.5	21.0
			99	VAPEEHPVLLTEAPLNPK	iTRAQ4plex@N-term; Lys->Gln@18		0.01	2097.14	700.05	2097.12	700.05	3		13.8	21.1	35.2	29.9
			99	YPIEHGIVTNWDDMEK	iTRAQ4plex@N-term; Methyl(E)@4; iTRAQ4plex(K)@16		-0.01	2248.10	750.37	2248.11	750.38	3		10.8	27.0	39.8	22.4
													Average:	19.1	21.6	31.8	27.5
													StDev:	8.9	11.7	6.4	10.6
