# Supplementary material for: Evolutionary Descent of Prion Genes from the ZIP Family of Metal Ion Transporters
Source: PLoS One. 2009 Sep 28;4(9):e7208. doi: 10.1371/journal.pone.0007208 (PMC2745754; doi:10.1371/journal.pone.0007208)
Supplement: Table S3 — (0.11 MB PDF) [file pone.0007208.s006.pdf]

Supplemental Table 3: The following sequences from Entrez, Ensembl and UniProt databases were utilized in the alignments.

SEQUENCES	Entrez	Ensembl	UniProt
**Ag_ZIP5/6/10**	XP_317935.4		
**Dm_ZIP5/6/10 (1)**	NP_001097608.1		
**Dm_ZIP5/6/10 (2)**	NP_523974.3		
**Dr_PrP**	NP_991149.1		
**Dr_Sho2**		ENSDARP00000074841	
**Dr_ZIP5**	XP_690258.2		
**Dr_ZIP6**	NP_001001591.1		
**Dr_ZIP8**		ENSDARG00000028214	
**Dr_ZIP10**	NP_956965.1		
**Dr_ZIP14**	XP_001340102.2		
**Ga_PrP-1**	CAL64057.1		
**Gg_PrP**		ENSGALP00000040285	
**Hm_ZIP12**	XP_002160698.1		
**Hs_PrP**		ENSP00000368752	
**Hs_ZIP4**		ENSP00000276833	
**Hs_ZIP5**		ENSP00000266980	
**Hs_ZIP6**		ENSP00000269187	
**Hs_ZIP8**		ENSP00000378310	
**Hs_ZIP10**		ENSP00000352655	
**Hs_ZIP12**		ENSP00000366588	
**Hs_ZIP13**		ENSP00000346956	
**Hs_ZIP14**		ENSP00000352779	
**Mm_PrP**	NP_035300.1		
**Mm_Sho**	CAF18554.1		
**Mm_Dpl**	AAF02544.1		
**Mm_ZIP4**		ENSMUSP00000073134	
**Mm_ZIP5**	AAH28990.1		
**Mm_ZIP6**	NP_631882.2		
**Mm_ZIP8**		ENSMUSP00000029810	
**Mm_ZIP10**		ENSMUSP00000027131	
**Mm_ZIP12**		ENSMUSP00000080911	
**Mm_ZIP13**		ENSMUSP00000073263	
**Mm_ZIP14**		ENSMUSP00000066108	
**Tn_PrP-1**			Q4SJ93
**Tr_PrP-1**	AAN38988.1		
**Tr_Sho1**	CAG34291.1		
**Tr_Sho2**	CAG34292.1		
**Tr_ZIP6**		ENSTRUP00000006592	
**Tr_ZIP8**		ENSTRUP00000045288	
**Tr_ZIP10**		ENSTRUP00000007404	
**Tr_ZIP12**		ENSTRUP00000019248	
**Tr_ZIP14**		ENSTRUP00000038591	
**Ts_PrP**			Q9I9C0
**Xl_PrP**	NP_001082180.1		

Ag → *Anopheles gambiae* (African malaria mosquito)
Dm → *Drosophila melanogaster* (fruit fly)
Dr → *Danio rerio* (zebrafish)
Ga → *Gasterosteus aculeatus* (three-spined stickleback)
Gg → *Gallus gallus* (chicken)
Hm → *Hydra magnipapillata* (hydra)
Hs → *Homo sapiens* (human)
Mm → *Mus musculus* (house mouse)
Tn → *Tetraodon nigroviridis* (spotted green pufferfish)
Tr → *Takifugu rubripes* (Japanese pufferfish)
Ts → *Trachemys scripta* (red-eared slider turtle)
Xl → *Xenopus laevis* (African clawed frog)
